# Foreign Summary

**Published:** 1840-06-13

**Authors:** 


### FOREIGN SUMMARY.

*Contributions to Surgical Pathology.* By WM. HENRY PORTER, Professor of Surgery in the Royal College of Surgeons in Ireland, &c.

*Aneurism of the Carotid Artery.*

In the details of the case which has given occasion to the following reflections, it may appear an almost fatal imperfection, that I have not been able to verify the correctness of my views by a post-mortem examination of the parts engaged in the disease, and perhaps the omission may, in some respects, diminish the interest that would otherwise attach to it; but although the case ended fatally, it was not in my power to procure permission to inspect the body, and the failure of my endeavours to obtain this object is one of the principal reasons to induce me to lay it before the profession. A length of time may elapse before I shall have an opportunity of seeing or treating an exactly similar case; perhaps (as they are very rare) I might never again meet with an example, and therefore I am anxious to call the attention of my brethren to the subject, in order that some one else may complete that which I shall only seek the merit of having commenced. There is another circumstance that invests this case with a more than ordinary degree of interest.—Although attended with several unpromising symptoms, I freely confess I undertook the operation with very sanguine expectations of success. After it had been performed, every thing seemed to progress very favourably; even when the unpleasant occurrence of a suppurating sac made its appearance, I was not much alarmed; but when death took place, from a cause that I had scarcely anticipated, and but slightly dreaded, it at once led me to reflect most seriously on the circumstances that could render an operation, generally so successful, in this particular instance so entirely abortive. The case to which I allude was one of aneurism of the carotid artery; the chief point to be verified by dissection, is, whether of the external or internal? I believe it was of the latter; and the reasons on which that opinion is grounded will be found hereafter.

In considering the causes that may interfere with or prevent the successful issue of an operation, and the recovery of a patient, it is rather an unusual view of the subject to regard the situation of the tumor as exercising a very decided influence, or to aver that the principle on which we treat an aneurism in the ham or in the thigh with almost a certainty of success, may not be applicable to the management of a similar affection in a different locality. Yet such, I fear, is practically correct; and although other collateral circumstances must be taken into account, as contributing their share in the production of this unpleasant result, yet I believe the internal carotid artery will be found so placed as to afford a peculiar facility for the occurrence of these untoward complications, and that operations performed for the cure of aneurisms of this vessel are attended with a degree of uncertainty scarcely to be expected elsewhere. In order to the correct understanding of this position, it will be necessary to say a few words on the manner in which an aneurism is cured by a ligature being placed on the trunk of the vessel, at the cardiac side of the tumor.

When such ligature is applied, it merely removes the impulse of the heart from the circulation through the vessel beyond it. The blood necessary to the nourishment of the limb or part at its distal side, is then conveyed thither by the anastomosing branches, which are generally too small to convey the impulse of the heart at the same time, and thus whatever blood enters the sac of the aneurism is no longer thrown in with a jet or *per saltum*. It thus can exercise no influence either on the fluid blood within the tumor, or on the elastic qualities of the sac or its coverings; it is allowed to remain quiescent within its cavity, and no portion of it is forced back into the general circulation again. So circumstanced, and at perfect rest, the natural tendency of the blood is to become coagulated, and the coagulum thus formed, being restrained by the sac and its coverings from extending itself in any other direction, is pushed towards the point where there is least resistance; that is, against the open vessel, which it compresses and causes to become obliterated, just as would happen to any bleeding artery under the continued pressure of a clot. In order, says Scarpa, that the compression may produce the union of the two opposite sides of an artery with each other, and at the same time the radical cure of the aneurism, it is necessary that, besides the vitality of the coats of the artery, the degree of pressure applied upon it be such as to place the two opposite parietes of the injured vessel in firm and complete contact and that it be at the same time capable of exciting the adhesive inflammation in the proper coats of the artery. Without the concurrence of these circumstances, the compression does not prove beneficial, or only produces an incomplete cure; for, whenever the compression is not sufficient to place the two opposite sides of the artery in complete and firm contact, and does not excite in them the adhesive inflammation, including also the root, properly speaking, of the aneurism, the canal of the lacerated or wounded artery remains open and pervious as before the use of the compression. If this view of the case be conceded (as probably it will,) it must follow that any thing capable of disturbing the blood within the sac will interfere with the process of coagulation, and thereby delay, if it does not prevent, the cure; and that any condition or position of the sac which will allow the pressure of its contents to be directed otherwise than against the injured vessel, must have a similar effect. Either of these circumstances taken singly, may prevent the cure; when both are combined, success is scarcely to be hoped for.

When pulsation is observed to return in the aneurismal tumor in a short time after the application of the ligature, it is obvious that the great object of completely cutting off the impulse of the heart has not been attained; that blood, to a greater or less quantity, not only enters the sac, but that some is returned again from it, and that the blood within it is constantly disturbed, and therefore kept in a state unfavourable to coagulation. This phenomenon of the reappearance of pulsation, has of late years been frequently observed, and many of the causes that may conduce to it are now well known ; some of them being of a nature entirely to prevent the cure of the aneurism, such as the existence of two large trunks in the limb, by an irregular arterial distribution, or where one or more large branches arise from, or otherwise communicate with the sac, and others which only delay the sanative process, but do not (at least necessarily) completely interfere with it. These latter are, firstly, the circumstance of the ligature being tied so loosely as not only not to stop the current of blood through the vessel, but not completely to cut off the impulse of the heart from the distal circulation ; and what is of more of importance, as bearing on the present subject, the existence of such an extensive and free communication by anastomoses as will convey to the tumor, by a circuitous route, the impulse which the ligature was intended to remove. I know not how far such a communication may be established in an aneurismal limb by a preternatural increase of size in the collateral vessels : such has been spoken of by authors, but I have no evidence of its existence in any one particular case to an extent sufficient to produce the phenomenon alluded to. However, I believe this unhappy condition obtains with respect to aneurisms of the external carotid artery in the neck, and that the free and extensive anastomoses through the vessels of the brain in their natural and normal state, will be quite sufficient, in some instances, as in the following case, to delay the process of cure; in others, perhaps, to prevent it.

On the 21st of August, 1829, I performed the operation of securing the trunk of the common carotid artery of the right side, in a woman of the name of Rourke, in the Meath Hospital. As this case has been already published in the fifth volume of the Dublin Hospital Reports, I shall merely remind the reader of some facts in connexion with it, which it may be necessary to refer to hereafter. It was a case of aneurism of the carotid, of fifteen years' duration, consequently its growth had been extremely slow, and it might be reasonably inferred that the aperture leading from the artery into the sac was very small; it was firm, hard, and solid, containing scarcely any fluid blood, and, on examining the fauces, no pulsation could be observed within. The progress of this case was attended with some unpleasant consequences, such as a return of pulsation in the tumor in four hours after the operation, and suppuration of the sac ; however, eventually my patient recovered, and left the hospital early in the following March, to resume her former occupation as a servant. She had never been a very healthy person, and was afterwards freqeuently an inmate of the hospital for pectoral complaints: however, at length she died on the 7th of September, 1836 ; and I may mention, as a singular instance of strength of mind overcoming the prejudices so constantly met with in persons of her condition, she bequeathed her body to me, and, by permission of the inspector of anatomy, it was sent to the school in Park-street.

On examining the neck it was recollected that, in the course of the operation, the sternal and large portion of the clavicular attachments of the sterno-mastoid muscle had been divided, yet, during the remainder of life, the patient never seemed to experience any inconvenience or imperfection of motion in consequence.—The condition of this muscle, then, first attracted attention. At the inferior part of the neck, and for two inches above the clavicle, there was not a trace of fibre remaining of the muscle, and its place was supplied by a dense and strong fascia attached to the clavicle below, and into which the remnant of the muscle was inserted above. In short, the inferior portion of the muscle seemed to have been converted into this fascia ; on dividing it, the jugular vein and the ligamentous-like substance into which the artery had degenerated, were exposed.

The remnant of the artery exhibited one continuous and unbroken cord from the bifurcation of the innominata to the division into internal and external carotids, so that, although the vessel must have been divided by the separation of the ligature, it had united again, and the exact spot at which it had been tied could not be ascertained. The internal carotid also was obliterated up to the spot where the ophthalmic artery was given off within the skull. In close connection with this, was the remnant of the aneurismal sac, a small, firm, fibrous tumor, of an oblong shape, and nearly of the size and form of a very large almond ; it lay a little below the posterior belly of the digastric muscle, and on the lingual nerve, to which it had some connexion, but not very intimate. The external carotid was pervious, but as compared with the vessel of the opposite side, greatly diminished in size, as were all its branches, excepting only the thyroid, which was pretty nearly of its natural dimensions. In consequence of the injection not being very perfect, the anastomoses were in general not minutely developed above the thyroid artery, but the communication between this and the branch ascending from the subclavian was extremely free, and the inosculations of these vessels within and on the surface of the thyroid gland very numerous, much more so than between the vessels of the opposite sides of the neck.

The subclavian on the right side was at least of twice the diameter of that on the left ; the vertebral was enlarged in the same proportion; and the ascending branch of the thyroid was also increased in size. The chief external communication was between the ascending and descending thyroid arteries, which seemed to be the medium by which the external carotid and its branches were supplied: the quantity of blood formerly brought to the brain by the internal carotid, was afterwards furnished by the vertebral, which became enlarged throughout its whole course, until the formation of the basilar artery, after which the entire circulation of the brain was perfectly normal.

This dissection proves that in this case at least, the re-appearance of the pulsation after operation, was caused bv the free and extensive anastomotic circulation through the brain, and that without the slightest appreciable enlargement of the collateral vessels.—The dissection was seen and examined by Dr. Hart, now Professor of Practical Anatomy in the Royal College of Surgeons in Ireland, and the preparation remains in the Museum of the School of Medicine and Surgery, Park-street. So far, then, it has been shown that the locality of the internal carotid is unfavourable to the cure of aneurism by operation, by at least delaying its progress; I have now to show how the same influence may prevent it altogether. But previously, it may be advisable to mention the leading particulars of a case in which such an unfortunate failure actually took place.

Matthew Markey, æt. 38, of low stature, and very strong make, admitted into the Meath Hospital, on the 19th September, 1838, with a very large aneurism, occupying nearly the entire of the left side of the neck. It extended from about three quarters of an inch above the clavicle to the mastoid process, was bounded posteriorly by the trapezius muscle, and anteriorly it pushed the larynx considerably to the right side. The entire circumference of the neck over the most prominent part of the tumor, was fourteen inches and three quarters: from the thyroid cartilage across the tumor, to the spinous process of the fourth cervical vertebra, nine inches and a quarter: from the same points, the measurement on the opposite side amounted to but five inches and a half. Examined by the mouth, the appearances of the tumor were most alarming: the pulsation could be distinctly seen, and the blood almost felt under the mucous membrane; it seemed ready to give way, and burst into the mouth every moment, and so remarkable, and so urgent was this symptom, that on requesting my friend Dr. Graves, then the physician in attendance on the hospital, to examine this patient, I received a note from him, strongly pressing the necessity of immediate operation, lest such a catastrophe should take place. It is needless to detail the other symptoms; they were such as are usually observed, except that the tumor was very soft, the blood within it evidently fluid, and, of course, the pulsation extremely violent. This peculiarity might, in some respects, be explained by the history of the case.

The disease may be said to have existed but a few days. Only five weeks had elapsed, since he first perceived a small hard tumor, like a kernel, near the angle of the jaw, perfectly moveable, without pain, and (as he stated) without pulsation. In the course of ten or twelve days, it became uneasy, but not actually painful, and he poulticed it, in the expectation that it would suppurate and break: it, however, increased in size, although slowly, and occasioned a good deal of annoyance in the motions of the head. It had then become distinctly pulsatile. Only seven days before admission, while at work, and after exerting himself considerably, he was suddenly attacked with most excruciating pain darting from the tumor across the forehead, and towards the vertex. He was immediately obliged to quit his employment and return home, where he discovered that the tumor had increased in size to a surprising extent, and that it pulsated with great violence. He suffered dreadfully for the next three nights, not sleeping, nor even being able to lay down his head. He was then attacked with hoarseness, which amounting at times to nearly a total loss of voice, alarmed him so much, as to cause him to apply at the hospital for relief, and he was admitted on the day above specified.

On the 22d of September, the operation of tying the common carotid was performed.—As the space between the tumor and the clavicle was extremely limited, I made a transverse incision at the root of the neck, parallel to, and above this bone, commencing internal to the superficial jugular vein, and extending forwards about two inches in length. Having thus exposed the sterno-mastoid muscle, I divided its clavicular attachment cautiously on a director, and came down on a very strong and resisting fascia, which, having slightly torn with the forceps, I also divided on the director. The edge of the sternohyoideus muscle could now be distinctly seen, which being carefully divided, I tore the sheath of the vessels partly with the nail of the fore-finger, and partly with the end of the director. The vessel was now exposed, and although the wound was deep, I could easily pass the needle I generally use, and of which I have given a description on a former occasion, round the artery, which was tied as tightly as I could draw the ligature. During the operation I experienced no inconvenience from the jugular vein: I might almost say I never saw it. I certainly saw the pneumogastric nerve, because I looked carefully for it; but the pleuri did not rise up in the neck, as I have experienced on other occasions. Altogether there was much less difficulty in the operation than might be anticipated; the patient bore it well: was but twenty minutes on the table, and walked up stairs to his ward afterwards, refusing any assistance. On the ligature being tied, the usual phenomena occurred; the pulsation ceased in the tumor; it became diminished in size; and the patient declared himself relieved from pain.

I had mentioned in my clinical lecture on this case, that I anticipated a return of the pulsation in the tumor at an early period after the operation, and the suppuration of the sac at one more remote. In the former of these expectations I was disappointed; pulsation did not return, although the tumor remained soft, and its contents evidently fluid. As to the full extent of this assertion, however, there may be some exception taken. Mr., now Sir Phillip Crampton, who took a great interest in the case throughout, always said that he perceived a weak pulsatile thrill in the tumor; and on looking at it in profile I sometimes saw, or fancied I saw, a slight motion corresponding with the action of the heart, like that which might be exhibited by a large swelling in the immediate vicinity of an artery; but on examining it with the hand, I never could feel a distinct pulsation; if such existed, therefore, it must have been extremely weak and indistinct.

It is unnecessary to enter minutely into the details of this case, which at first appeared to progress as favourably as could be desired. On the fifteenth day after the operation the ligature came away with the dressings; and on Saturday, the 20th October, exactly four weeks after the vessel had been tied, I find the Hospital Report to be as follows: "Patient's health is now very good; he is up all day and walks about the grounds; sleeps well during the night; has no pain or uneasiness; the discharge from the wound daily diminishing in quantity, and assuming a more healthy character." But on Monday, the 22d, matters began to assume a different aspect. He complained of pain and stiffness in the neck, with headach, furred tongue, and general constitutional derangement. The sac had begun to inflame. On Saturday, the 27th (five weeks after the operation,) "the pain and stiffness in the neck had greatly increased; the skin tense and shining, of a deep red colour over the centre of the tumor, more faint towards its border; the apex soft and elastic, with a distinct sense of fluctuation: he described the pain as being most excruciating, and of a hot and throbbing character. He had intense headach; foul tongue; bowels obstinately costive; pulse 96, hard and full. He also suffered from constant cough, difficult respiration, and painful deglutition. The sac had suppurated, but as this was an occurrence which had frequently taken place in my experience before, I acknowledge it occasioned me little uneasiness, and I prepared to treat the case in the manner I had treated others with uniform success.

I made a free incision into the tumor with a view to discharge the matter, turn out all the coagula, and then, by applying pressure externally, seek the obliteration of the sac. The incision gave exit to a large quantity of pus mixed with *fluid blood*, and I found I had opened into a large cavity which scarcely contained any coagulum at all. I laid down the sides of the wound, and endeavored to apply pressure by means of compresses retained by numerous straps of adhesive plaster. This latter indication, however, could not be accomplished. Direct pressure caused an intolerable sense of suffocation, and the consequence was, that under the moderate degree employed, the sac suppurated freely, and the discharge became profuse. Still I imagined I had no cause for apprehension beyond the wearing and wasting hectic that would probably be established; and was therefore surprised at being called at 3 o'clock on the morning of the 30th, with information that my poor patient was bleeding profusely from the wound I had made in opening the abscess. I hastened to the hospital, and found him literally bathed in blood; and, notwithstanding the exertions of a most active and intelligent pupil who was resident there at the time, he must have lost between forty and fifty ounces. The bleeding was kept under by the pressure of this gentleman's hand, but immediately on its being removed, the blood spouted forth with considerable force. On examining the cavity, with the aid of a very imperfect light, I discovered several streams of arterial blood passing in different directions through a broken clot at the bottom of the sac, and my first impression was, that some branches opened into and communicated with the cavity; and as the man must have died of hæmorrhage, whilst I should be endeavouring to secure these I determined on trying to place the patient in the same condition as if the sac had never been opened, and trusting to the pressure of the coagulated blood for the suppression of the hæmorrhage and the obliteration of the vessels. I therefore passed four needles through the lips of the wound, and applied the twisted suture, which held them firmly together, and effectually stopped the bleeding, and I left my patient safe for this time, but pale, weak, sunken, and evidently unable to bear the loss of more blood.

Friday, the 2d October. The blood burst out again, welling up profusely from the bottom of the wound, but coming without impetus. As I had now the advantage of the assistance of my colleague, Mr. M. Collis, I determined to explore the cavity, with the view of securing the bleeding vessel or vessels, and if possible preventing any further loss of blood. I opened the full extent of my incision, and began to clear out the cavity, when the blood burst forth in a stream, equally frightful and uncontrollable, flowing from a rent in the vessel that my finger could not cover. In this predicament not a moment was to be lost. The patient had lost so much blood that a single minute would probably decide his fate. As for seeking to tie a vessel lying at the bottom of an enormous cavity, and fully at a distance of five inches from the surface, it appeared to be wholly out of the question; and the vicinity of the pneumo-gastric nerve and the deep jugular vein rendered a plunge of the needle or the employment of the actual cautery equally objectionable. I had no resource but to fill this enormous cavity with compresses of sponge, which should be maintained in their places by closing the integuments over them, and this latter could only be effected by means of needles and the twisted suture. Straps of adhesive plaster were totally useless; glue spread on leather was tried, and found equally inefficacious; nothing remained but to stitch the wound in the manner specified, and it certainly had the effect of perfectly restraining the bleeding for the time being. However, I knew that there could be no permanent benefit derived. I knew from the examination I was enabled to make, that the blood proceeded from the original aneurismal rent, that the artery which was not affected by the usual process of nature during the five weeks that intervened between the operation and the suppuration of the sac, would scarcely become obliterated under the pressure of the sponge, and that the needles must ulcerate the parts and cut their way out long before any permanent benefit could be achieved. Immediately on the wound being perfectly closed, the pulsation, which had disappeared, or been so weak as to be only perceptible to the most delicate touch, returned in the tumor as vividly and as violently as before the operation had been performed at all.

It would be tedious to dwell on the minute reports of this awful and melancholy case; suffice it that the patient still continued to bleed at intervals. According as a needle would separate, or a compress be disturbed, blood would burst forth with greater or less violence, and although the hæmorrhage was always promptly restrained, yet these successive losses, in conjunction with pain, loss of sleep, and extreme anxiety, reduced him so low, that he expired without a struggle, on the evening of the 12th October—having lived thirteen days from the first appearance of the bleeding. Every effort was made to procure a *post mortem* examination, but in vain; and I have been left to speculation to account for the singular phenomena and unexpected results that attended this most interesting and important case. But it is not altogether without a parallel; and, after some research, I have discovered a case which will throw additional light on these cases of aneurism of the carotid artery.

On the 15th April, 1831, Mr. Green, in St. Thomas's Hospital, tied the common carotid for the cure of an aneurism, situated (as he believed) at the point of its division. The patient was a man advanced in life, being 65 years of age: the tumor had existed from the preceding Christmas, was slow of growth, and had attained only to the size of a walnut; in other respects the symptoms detailed bear a strong resemblance to those observed in the case just related. On the ligature being tightened, a manifest and instantaneous diminution of size took place in the tumor and in the force of pulsation, which was yet distinguishable; but the patient having been carefully removed to bed, in about an hour this had ceased altogether. It is worthy of remark, that doubts were entertained by many who carefully observed the case as to whether the pulsation ever ceased completely.

"On the twenty-fourth day the ligature separated, the noose thereof being perfect and firm, and the dressings having been applied to the wound daily. This has some exuberant granulations, not occupying more than half an inch, which have been touched with argentum nitratum. A feeble pulsation has been constant since the 20th April, (the fifth day after the operation,) and we are of opinion that it has latterly been more vigorous; the tumor itself is very materially diminished, but not to the degree that, at this distance of time from the obliteration of the canal of the vessel, we should reasonably expect."

"May 31st. Arterial pulsation has become more distinct in the tumor, but is yet weak. It is supposed, from the situation of the latter, (at the bifurcation of the common carotid,) which is favourable to such, that a communication exists in that part, between the external and internal divisions."

"June 14th," (two months after the operation.) "The aneurismal sac has grown larger within the last fortnight, and the pulsation remains equally, if not more powerful."

"July 28th. Patient is suffering from suppuration of the right tonsil; a diffused swelling has taken place externally upon the neck, a little below the angle of the jaw. The tonsil was opened, and discharged pus freely."

"September 20th. The above local symptoms subsided in due course, but the aneurismal swelling retains the same trifling pulsation."

I have merely extracted, from the printed report of the above case, the points in which it bears some resemblance to that of my patient, and which seem to have some reference to the locality of the disease. It was supposed by Mr. Green to exist at the bifurcation of the carotids, a spot equally favourable with the internal itself for the return of pulsation through the medium of the cerebral circulation.

This pulsation did return; and, according to the opinion of some, never totally disappeared. About the 28th July, symptoms occurred which might have been occasioned by the suppuration of the sac, and at the end of five months pulsation remained;—the disease had not been cured. The case seems to hold a middle place between that of Elizabeth Rourke, in which the progress of the disease was extremely slow, and the contents of the sac nearly solid, and that of Markay, which had proceeded so very rapidly, and in which there was probably no coagulum at all. It is to be regretted that the appearances of the tumour, in relation to the mouth and pharynx, were not described, and its ultimate result not ascertained.

Thus far, however, I have endeavoured to show, that aneurisms of the internal carotid are unfavourably placed for the accomplishment of one part of the cure; that a ligature on the trunk does not entirely cut off the impulse of the heart from the diseased vessel; that such impulse may be conveyed through the circulation of the brain in its normal state; and that the effect of this impulse must be, to disturb the blood within the sac, and delay, if it does not prevent, its coagulation.

I have now to direct my attention to another circumstance of at least equal importance. In a limb, when the aneurismal sac becomes distended with blood after operation, the pressure exercised thereby must be directed against the injured vessel; the structures external to and surrounding such sac are, many of them, inelastic, all more or less resisting. They will not permit a growth or extension of the tumour in any direction towards them; and, consequently, when the sac is filled, and more particularly when its contents are solidified, it must not only press against the ruptured vessel, but compress it to an extent and degree to occasion its obliteration. But with respect to the internal carotid the case is widely different. However covered with fascia, and muscle, and skin, and other resisting structures externally, it is wholly unprotected in the direction of the pharynx; for, on making a vertical section of the head and neck, and dissecting from within outwards, I find that the internal carotid is very close to the pharynx. In its passage upwards, from the bifurcation to its entrance into the skull, it obliques slightly backwards and inwards, having behind it the sympathetic nerve and first cervical ganglion, where it rests against the spine: external, it has the styloid process, the three styloid muscles, the digastric, the mastoideus, and the different layers of fascia; a little in front it has the stylo-pharyngeus muscle, but internally, or towards the pharynx, it has nothing but the mucous membrane, the contrictor of the pharynx, some very loose cellular tissue, and the twigs of the superior laryngeal nerve; thus the aneurismal sac has ample room to grow and increase inwardly, and, consequently, the pressure it is forced to make on the opening in the vessel may be so trifling as not in any way to lead to its obliteration. This circumstance may explain (although in Mr. Green's case it does not appear that the mouth and pharynx were ever examined) why that case proved to be what Scarpa might term an imperfect cure; and why, in the case of Markay, in five weeks after the vessel was tied, and the direct force of the heart cut off, there should have been no advance whatever in the process by which the artery might be obliterated. It appears curious that the position of this vessel, with respect to the pharynx, should not have attracted more attention in the examination of aneurismal tumours; for, reasoning from the anatomy of the parts, I should be disposed to believe that the symptoms of pulsation would always be most clearly observed from within. It was so in the present case; and I have since had an opportunity of seeing it under circumstances where it might be still less expected or looked for.

A young girl, named E—— M——, was admitted into the Meath Hospital, with a varicose aneurism, situated at the angle of the jaw, and extending downwards a short way along the course of the external jugular vein. It became very large and prominent when pressure was made on this vessel below, so as to interrupt the current of blood; it became then excessively painful, and exhibited the usual thrilling sensations both to the finger and the ear; but when looked at from the mouth, a strong and continued pulsation, together with considerable tumefaction, was obvious to every eye. This disease had been produced by a stab of a scissors, inflicted seven years before, and no very decisive treatment was adopted for it in hospital. In fact, the exact nature of the lesion was not understood: and it was only matter of conjecture, that a communication had been established between the external jugular vein and the internal carotid artery, with the intermediate existence of a varicose aneurismal sac. The case, however, is pathologically interesting, as affording an illustration of the facility with which such tumours may grow and increase internally, or towards the mouth.

Whilst on this subject, I may be permitted to notice a circumstance in the case of Markay, in explanation of which I confess myself unable to form even a remote conjecture. Why did the blood remain in a fluid state within the sac during five weeks? Before the artery was tied, and whilst a large current was forced into the sac with the full strength of the heart's action, it is not difficult to conceive that such constant and violent motion might interfere with the process of coagulation; but that after such an interval, the blood, with the exception of one or two very small coagula at the bottom, should have been found perfectly fluid, was scarcely to have been expected, and must have had a very unfavourable influence on the success of the operation. Yet such was the case, and it is no easy matter to explain why it should have been so. But the difficulty of solution attendant on this question leads me to propose another. Is there a pathological difference in the blood of different individuals, giving to that of one a greater or less tendency or disposition to coagulate, than to that of another? If there is such a difference of condition or constitution, a knowledge of the fact, and more particularly of the causes or circumstances that lead to its production, might prove of incalculable benefit in the management of disease in general, but more particularly those of the circulating system. And that there is such a difference I am strongly disposed to believe, although being totally unprepared with satisfactory proofs, I dare not offer it even as an hypothesis—but rather as a suggestion that may lead others (as opportunity may offer) to investigate the pathology of the blood as promising to lead to invaluable practical results. That the blood of individuals suffering from different diseases will exhibit different phenomena in the quickness with which the coagulum is formed, and the degree of firmness and solidity it reaches, no one will be disposed to deny; but the point to which I wish to direct attention is, that the blood of a person apparently in a healthy state may not coagulate under circumstances wherein that of another individual would almost certainly do so.

Some years ago, a man was admitted into Meath Hospital, having received a stab of a sharp-pointed shoemaker's knife about an inch below the right sterno-clavicular articulation, by which the internal mammary artery and vein were wounded. These vessels poured out their blood continually, and in such abundance, into the cavity of the pleura, that the lung became dreadfully oppressed, and it was deemed advisable to perform the operation of paracentesis on the fourth or fifth day after the receipt of the injury. The wound made in the operation was large, in the expectation that it might facilitate the escape of any coagulum; but the precaution was found to have been unnecessary, as the blood had remained in a perfectly fluid state, and flowed away with the greatest facility. The quantity of blood thus lost was enormous: it must have amounted to some quarts: and as the wounded vessels still continued to bleed, it is scarcely necessary to add that the patient soon died. On examination after death, a good deal of fluid blood was found in the cavity of the pleura, but not a particle in the state of coagulation. Is it not reasonable to conclude, that in this case there was some peculiar condition of the blood that rendered it incapable of coagulation? and might not this have been one cause of the continued and unceasing hæmorrhage, without the slightest effort on the part of nature to arrest it?

On the 5th August, a man named James Wilson was admitted into the Meath Hospital, with an enormous aortic aneurism.

Three months previously he had perceived a tumour of the size of a hazel-nut above the clavicle, and close to the sternum, which tumour increased in size daily, enlarging from below upwards: on admission it occupied the anterior and right side of the neck, extending as high as the thyroid cartilage, and slightly displacing the larynx. It pulsated violently. From the symptoms, the history of the case, and careful stethoscopic examination, it was decided that it was an aneurism of the aorta, and that palliative measures only should be adopted. He remained in hospital until the 8th September, when the tumour burst, and the patient died in a few seconds, with an awful gush of blood.

*Post mortem examination.*—On looking at the situation occupied by the tumour during life, it appeared to have shrunk and collapsed, and instead of being elevated, there was a very large cavity, in the centre of which was a dark spot marking the place at which it had given way. The sac was eight inches in its long diameter, by five in the transverse at its widest part, and sprung from the aorta at the root of the innominata, passing up behind the clavicle, which was in part carious. Not a particle of lymph, or fibrine, or coagulum of any description, existed in the cavity of this immense sac, although upwards of four months had elapsed since the commencement of the disease, and the tumour had not increased with any very extraordinary rapidity.

From these cases, and from other facts and observations, with which it is unnecessary to swell this paper, already pushed to perhaps a tedious length, I have been led to believe that a condition of blood indisposing to coagulation, may exist in individuals otherwise apparently healthy, and exhibiting no symptom of such an abnormal state. If such an opinion be correct, and if (as is generally conceded) the coagulation is an active agent in the suppression of hæmorrhage, and a necessary part of the process in the cure of aneurism, few subjects can be submitted to the attention of the pathologist of greater apparent importance, or more likely to repay the trouble of investigation.—*Dublin Journal for March.*

---

*On the Sympathy between the Cerebellum and the Testes.* By Dr. J. Budge, of Altenkirchen.—Gall placed the organ of the sexual appetite in the cerebellum; but though the attention of physiologists has been, since then, repeatedly directed to this subject, they have not yet arrived at any definite result. For, even if we collect all the known cases of diseases of the cerebellum, as Burdach has done, we shall find, that, though an actual affection of the sexual organs has occurred in no small number of the cases, yet that in the greater number no such affection existed. It appeared to Dr. Budge, that a more certain and incontrovertible kind of proof, viz. that of experiment, was wanted; and he thinks that he has at length succeeded in demonstrating by experiments on numerous animals, that this influence of the one organ upon the other is of the most simple, distinct, and certain nature.

Old cats were the animals selected, as those best adapted for experiment, and the effects were observed both during life, and immediately after death. The experiments were repeated so often that there did not exist the least doubt in regard to the results; and though in some animals the phenomena were far more distinct than in others, yet they were in all so similar that the narration of one may serve to illustrate what took place in all.

In a twelve year old cat, which was killed by wounding the heart, the skull was removed as quickly as possible, the abdominal cavity opened, and both testicles with their spermatic cords and *vasa deferentia* exposed. Not the slightest motion was observed in the testicles. The cerebellum was now stimulated with the point of a knife; and this had scarcely been done for more than three seconds when one testicle raised itself up, and moved from the spermatic cord on which it had lain, so as to form nearly a right angle with it; at the same time it became more and more tense. The more the cerebellum was irritated the more the testicle moved. The cerebellum was stimulated here and there, but the two testicles never moved at once. The cause of this, however, was soon discovered. When the right lobe of the cerebellum or the right commissure were stimulated, the left testicle always moved; when the left lobe and the left half of the commissure were stimulated, then as regularly the right testicle moved. The movements of the testicles were thus completely under control, and the stimulation of the cerebellum caused motion in these organs fully half an hour after life was apparently extinct.

Dr. Budge is of opinion that the cerebellum is the part where the nerves of the testicles have their terminal point; that they cross each other in the brain as those of all the rest of the body; and that they must lie tolerably superficial in it, because irritation of the deeper parts of the cerebellum did not succeed in producing motion of the testicles. He thinks it even probable that the union of the nerves takes place in the region of the first cervical vertebra, because stimulus of this part of the cord is very often accompanied by erection and discharge of semen, as in the hanged, &c.—*Edinburgh Med. and Surg. Journ., from Muller's Archiv.*, 1840.

---

*Congenital Hernia of the Diaphragm, with a portion of the Small Intestines in the Left Thoracic Cavity.* By Dr. Murphy.—M. C., after a labour of moderate duration, was delivered of a still-born child. There was no difficulty in the labour, nor any apparent cause for such a result. The heart was pulsating strongly, rather to the right side, but there was not the slightest attempt at respiration, and every effort to excite it failed. The child was rather above the average size; both wrists were distorted, and it had *spina bifida.*

On opening the thorax and abdomen, the cause was at once explained. The whole of the left thoracic cavity was filled with the small intestines, the development of the lung on that side having been arrested almost at its commencement. The heart was larger than usual, but nearly in its natural situation. The right lung was of the ordinary size, having the inferior lobe in close contact with the supra-renal capsule. The diaphragm was deficient posteriorly, but much more so on the left than on the right side.—*Dublin Journal.*

---

*On the Adulteration of the Sulphate of Quinine.*—The recent rise in the price of sulphate of quinine has induced many unprincipled venders of drugs to adulterate it with various ingredients; to such an extent, indeed, has this been carried, in some instances, that not more than a fifth part of what was sold as sulphate of quinine really consisted of that substance. M. Vallet found that the substance chiefly used for the adulteration of the sulphate of quinine was Mannite, a substance similar in external appearance to the sulphate of quinine, but destitute of all the valuable properties for which quinine is so justly celebrated. He found, however, that the adulteration could be with great ease detected by means of pure alcohol, which dissolves the quinine alone, but leaves untouched the mannite, which, however, is freely soluble in water and of its characteristic sweet taste.

M. Dubail has also arrived at the same conclusions as M. Vallet, and, in pursuing his investigations, met with one sample of sulphate of quinine, which, though presenting all the external characters of the genuine unadulterated drug, both as regarded its lightness and silky appearance, did not contain above a sixth part of its weight of sulphate of quinine. The rest was composed of mannite, insoluble in alcohol, but soluble in water, and of a sweet taste.

M. Pelletier has found that that which is sold in sealed packages, with the impress of his own seal, has also been subjected to adulteration, but the substance used in this instance, is gypsum. The same test, however, viz. the solubility of alcohol, applies to this case, so that every druggist would do well to apply this simple test to the samples of this drug before purchasing.—*Edinburgh Med. and Surg. Journ., from Journal de Pharmacie*, January, 1840.

---

*Successful Blepharoplastic Operations.*—Several interesting cases of restoration of the eyelids by operation have been lately related in the German journals; and, as the operation is relatively new to this country, the following cases cannot fail to interest.

1. A healthy man, of about thirty years of age, observed a small fissure on his left lower eyelid, near the internal angle, which appeared there without known cause. He paid no attention to it at first, but after some little time it inflamed, became covered with a thick crust, beneath which appeared an extensive ulceration; the conjunctiva inflamed, and ectropium followed. Various curative means were employed without effect, and even excision of the diseased portions did not stop the progress of the malady. The patient then applied to Professor Beck, who found the ulcer presenting a bleeding extremely painful surface, a malignant aspect, and having all the characters of a cancerous ulceration; it occupied the internal angle of the eye, a part of the *caruncula lachrymalis*, and had destroyed the whole of the lower eyelid. The complete removal of all the diseased portions, and the consecutive restoration of the eyelid according to the method of Dieffenbach, appeared to be the only plan which promised success. The operation was, therefore, performed in the following manner:—Two incisions were made at the commissures of the eyelids, above the diseased portions, and the upper eyelid dissected back to a small extent. Two other incisions from these angles were then carried downwards, so as to unite at an acute angle on the cheek, and include the whole of the diseased mass, which was then carefully dissected out. A horizontal incision was then carried from the external angle of the eye towards the temple, to an extent equal to the length of the eyelid which had been removed, and a triangular portion equal to the size of that which had been removed with the diseased mass, was cut out from the lower surface of the horizontal incision. On being carefully dissected back, and turned round, it was found to fit exactly the site of the lower eyelid and diseased mass which had been removed. The flap was kept in its place by sutures; and in a few weeks the union was complete, and the cicatrization of the wound on the cheek scarcely perceptible. It was then observed that the external palpebral commissure was so tense that the eyeball could not be sufficiently uncovered, unless the patient pulled down the artificial lower eyelid with his fingers. To remedy this defect, an incision was carried for a little way across the commissure in the line of the opening of the eyelids, and the conjunctiva drawn forwards, and attached by suture to the upper surface of the incision, so as to make this portion part of the upper eyelid. A similar portion of conjunctiva was also drawn forwards, and fixed by suture to the lower half of the incision; and after cicatrization the eyelids presented their usual appearance both with regard to their extent and direction. The lower eyelid did not become inverted, but remained free and soft at its upper or free margin, and did not incommode the eyeball in its motions.

2. A small tumour developed itself during the summer of 1835 on the free margin of the right upper eyelid of a scrofulous infant of nine years of age. It rapidly grew larger, became the seat of sharp, lancinating pains, and the palpebral conjunctiva became inflamed. At the end of a few months this tumour had attained the size of a walnut, became the seat of an unhealthy ulceration, which discharged blood and fœtid icherous fluid. No doubt remained as to the disease being of a cancerous nature. The base and the internal fourth of the eyelid were sound, but externally the turgescence extended to the palpebral commissure. Professor Schwærer operated in the following manner:—The diseased mass was included between two incisions carried round it, and entirely removed; and from the external angle of the eye on the external side of the eyebrow, a flap was dissected off, leaving it connected by a slender neck, and being turned round was carefully applied to the space from which the diseased mass had been removed and fixed by sutures. Adhesion took place by the second intention. After the adhesion was completed, the new eyelid appeared to be well formed, and was moveable at will.

3. A man, fifty-four years of age, had for ten years suffered from venereal affections, which had produced ulceration of the bones of the face and various ulcers which had considerably injured the left eye. The whole of the under eyelid was destroyed, with the exception of a small portion on the internal angle, which was, however, completely turned outwards, and exposed the whole of the conjunctiva. The cicatrices had also drawn down the upper eyelid at its external margin. The eyeball was in a state of constant inflammation. To remedy this, Dr. Burrow of Konigsberg performed the following operation. After having removed a considerable portion of conjunctiva, which was altered in its texture, and presented a bleeding surface extending from one angle of the eye to the other, he dissected from the cheek a flap, the pedicle of which was attached on the side of the nose, and applied it accurately to the pared surface, and fixed it there by three twisted sutures. The cure was completed by the fourteenth day, and the new eyelid fulfilled completely the end proposed.—*Ib.*, *from Ammon's Monatschrift*, Bd. i. Heft i. 1839.

---

*Extirpation of the Clavicle.* By Professor Regnoli, of Pisa.—A man of about thirty-four years of age, when lifting a sack of grain, was seized with a sudden pain in the left shoulder, which ceased in a short time, but returned whenever he attempted to resume his work. Ten days after the accident the pain had become constant, and was so much increased in severity as to deprive him of sleep. The shoulders and surrounding parts swelled and inflamed, and, in spite of appropriate remedies, matter formed, and found its way to the surface, through several ulcerated openings. About six months after this he applied to the hospital at Pisa. At this time a large ulcerated surface, with thickened margins, extended over the greater portion of the clavicle, and discharged a considerable quantity of unhealty sanious matter. When a probe was introduced to the bottom of the sore, the clavicle was felt denuded, and more minute examination detected that the greater portion of this bone was in a state of necrosis. The patient was very much reduced, and in a constant state of febrile excitement. Free incisions were made over the diseased bone, and other appropriate remedies pursued; but as none of these seemed to give relief, and as the emaciation and fever were progressively increasing and wearing out the patient, it was resolved to remove the diseased clavicle by operation.

The clavicle was laid bare its whole length, when the central portion was found to be completely isolated, and was removed with the forceps. The sternal extremity being also necrosed, was carefully disarticulated and removed, but as the acromial extremity was apparently healthy, it was left. The whole of the neighbouring parts were much altered in appearance from the effects of the inflammatory processes to which they were subjected. The wound was filled with charpie, and, though the process of cicatrization was slow on account of the weak state of health, yet it went on progressively,—several portions of the acromial extremity of the clavicle, which had been left, came away during the suppurative process. When the cure was completed, a fibro-cellular structure was found to have occupied the site of the clavicle which had been removed. The patient could move his arm in all directions, and use it for most purposes. It was, however, still somewhat weaker than the other arm, and had not yet perfectly recovered its muscularity.—*Ibid. From Annali Medico-Chirurgici di Roma*, June, 1839.

---

*Case of Poisoning by Cheese.*—A father, with his two children, their servant, and two other young persons, partook of about two ounces and a half of a particular kind of cheese, called in the country *knapost.* (It is made of curdled milk, which is allowed to stand till worms are developed in it.) At the end of about two hours, these six individuals were seized with the most alarming symptoms; sickness, tremors, sensation of burning in the throat and stomach, vomiting, purging, blue coloration of the skin, coldness of the surface, cramps, and faintings. Emetics of ipecacuan, with infusion of chamomile, were administered, followed afterwards by antispasmodics, and spirituous frictions over the epigastrium. The symptoms abated in from six to ten hours in the various individuals; but weakness remained for several days.

Chemical analysis failed to discover any trace of either mineral or vegetable poison. A concentrated decoction of the cheese, however, had an excessively acrid sour taste, which left a sensation of burning on the tongue.

The cows which furnished the milk for the preparation of these cheeses, pastured in a meadow rich in alliaceous plants, (probably the *Allium scorodoprasum,*) and the natives of the district say that they are very subject to diarhœas and hematurias.—*Ibid. from Schmidt's Jahrbucher, t.* 21, 1839.

---

*History of a Cæsarean operation in a twin pregnancy.* By Dr. Scholler, of Berlin.—The subject of this operation was a poor woman of the middle stature, 40 years old, and affected with lateral curvature of the lumbar portion of the vertebral column. She had suffered severely in early childhood, from rickets, and had not been able to go alone till after her fifth year. Afterwards she had had constant good health; she menstruated first after her twentieth year, and had continued since to do so regularly. In 1836, while unmarried, she became pregnant, and was received into the obstetric department of the hospital, where, in 1837, after a labour of two days and a half, with a presentation of the right shoulder and arm, she was delivered by turning and extraction of a dead and putrid child, the bones of whose skull, separated from their mutual connections, lay loose within the integuments of the head, as in a sac, containing a thickish pulpy substance that presented no trace of brain. In three weeks after her delivery she left the hospital perfectly well.

A year after this time she married, and soon became pregnant again, but again escaped the danger that she must have encountered from the delivery of a full grown and living child, by an accidental abortion in the fourth month. In the middle of February 1837, she again conceived, and this time her gestation was carried to the full period. She first had labour-pains on the 17th of November, in the evening, and they continued through the night, though they were neither severe nor frequent. During the 18th, two mid-wives and a surgeon made ineffectual attempts to deliver her, and at five in the evening she was brought to the hospital.

Examination detected a very narrow conjugate diameter of the aperture of the pelvis, which amounted at most to two inches and a quarter. Near the umbilical cord, which had protruded for some time from the os uteri, and was now pulseless, the head was felt lying quite above the brim of the pelvis, of which the external measure, by Baudelocque's compasses, was six inches. The furrow between two unequal bulgings of the distended abdominal walls gave the idea that she was pregnant with twins, and auscultation proved, in the most striking manner, the existence of a child still living, together with the child which, as its umbilical cord had been protruded beyond the vagina and pulseless for five hours, was certainly dead. The Cæsarean operation, therefore, was now deemed absolutely necessary, and it was commenced at ten o'clock at night, and performed by Dr. Busch, in three minutes and a half. An incision, about six inches long, was made in the linea alba, beginning just below the umbilicus, and extending to within an inch and a half of the symphysis pubes. On opening the abdominal walls, which were about two lines thick, some spoonsful of yellowish serous fluid flowed out. On cutting through the walls of the uterus, which were about five lines in thickness, and in which the incision was five inches long, a considerable hæmorrhage took place, and the knife came, as we expected, directly upon the placenta: the operator turned the latter off to the right side, and the arm of the dead child presented. It was seized by the feet (as they should always be) and drawn out, and its umbilical cord was divided.

The membranes of the second child were now seen to the upper and left side of the wound, with a dark, bluish, glistening aspect. They were broken, and the child, in the same way as the first, was drawn out by its feet. It soon cried out, and was given to the care of the attendant midwife. The separated placentæ were then taken away and removed with the membranes. The protrusion of the intestines, which would now so easily have taken place in the rapid contraction of the uterus, was entirely prevented by pressing on the abdomen. The wound was united by sutures, and lightly dressed with plasters and oiled compresses, and the patient was carefully put to bed.

For the first two days after the operation the patient went on satisfactorily, though with many signs of acute peritonitis; after that time, however, she began to sink, and, notwithstanding the judicious treatment which is detailed at some length, she died on the morning of the 23d, four days and a half from the performance of the operation.

On opening the body, twenty-four hours after, the large intestines were found adherent by lymph to the omentum, whose lower edge was similarly connected to the abdominal walls, and to the uterus at its upper part, by a broad thick band of lymph. Some adhesion of the intestines to each other were also observed, but nothing like a generally diffused intestinal inflammation. On the left side, near the descending colon, there was a serous exudation, and on the right, just below the cæcum, a dark coagulum of blood adhering to the abdominal wall. The abdominal organs generally were healthy. The uterus was as large as the head of a new-born child. Its peritoneal covering in the neighbourhood of the wound, which passed through only the body of the organ, and did not extend nearly to the fundus, was thickened; the wound itself was open, and its everted edges were discolored; the apertures in the large blood-vessels were filled by bright-red coagula, which passed far into their canals. The situation where the placenta had been attached was rough and shaggy; there was a coagulum at the base of the uterus, and some distinct remains of decidua still adhering. The wound in the abdominal walls was found completely united on its under surface, by a layer of plastic lymph effused over it; on its external surface it was united only at one point.—*Lond. Med. Gaz. from Medicinische Zeitung*, Jan. 1840.

---

*Efficacy of cold water, in the form of a descending douche, for old ulcers of the feet.* By Dr. Butzke, of Schwetz.—Atonic ulcers of the feet are very difficult to cure. They may continue without any internal cause, from a local secretion having become necessary to the system, or from the diminution of vital energy, or from organic degeneration of the skin and cellular membrane in the vicinity of the diseased surface. Dr. Butzke has succeeded in curing these ulcers easily and perfectly by the cold douche, which, by its enlivening and astringent power, removes the local atony of the skin round the ulcer; and this without repose being rigidly enforced, and without purgatives or limited diet. The method adopted was as follows: The water was brought from a spring into a wooden cistern four feet long, two broad, and two deep. On one side of the cistern, just above its bottom, were four wooden pipes, out of which the water fell from a height of six feet, in a strong unbroken stream. A bench was placed in front of the cistern, and a small footstool under the streams of water. The patient sat astride the bench, so that only the diseased foot, which was placed upon the footstool, was touched by the water. The douche was generally applied for half an hour, or in ulcers of a very bad kind, for an hour, every afternoon, without regarding the weather. When the douche was over, the foot was wiped dry and bandaged, the ulcer being merely covered with charpie.

The first effect of the douche was acute pain in the ulcer, which was sometimes so violent, that it was necessary to discontinue the application in ten minutes; then there came on a dark phlegmonous redness of the skin near the ulcer, and occasionally a slight hæmorrhage from the sore. When the douche was over, the secondary effects were a peculiar crawling and itching on the surface of the ulcer, considerably increased heat, swelling, and a rosy colour of the surrounding skin, together with the secretion of a thin lymphatic pus from the ulcer, and an increase of perspiration in the diseased extremity. The douche was less efficacious in herpetic ulcers of the feet, and scrofulous caries of the lower extremities; yet even in these cases one-half of the patients were cured.—*Lond. Med. Gaz. from Med. Zeit.* and *Schmidt's Jahrbücher*.

*Effects and Mode of Application of Remedies.*

*Suppositories.*—When substances are introduced into the rectum, by the simple instrument, consisting of a tube and moveable rod inside, very different effects may be expected from the same when introduced *more majorum* by the finger. In the former case it is lodged above the sphincter, and much less mechanical disturbance accompanies it than in the latter, which is in all respects a detestable process. In irritation of the bladder, or painful affections of the uterus, two grains of the watery extract of opium, with six of soap—or, in spasmodic states of the uretha, a grain of extract of belladonna, are attended with marked benefit, and are productive of no inconvenience. It has been an old established practice among nurses, in case of refractory children, to introduce a fragment of soap with the finger, and generally, the necessity of giving purgatives is in this way for the time avoided; but on consulting authors, I find no account of purgative suppositories for adults, except some intended to cause the expulsion of ascarides from the rectum,* since the time of Hippocrates, who is said to have composed them of salt and colocynth.

Being desirous to ascertain to what extent purgatives would act, when introduced, into the rectum in the form of suppository, I in the first instance fixed on assafœtida, and ordered it to be introduced by the tube and rod in eleven cases. It succeeded in all, producing one or two dejections, except in two, in whom it failed, and in one individual, who suffered much from flatulent distension of the colon, it was attended with relief much superior to that afforded by other purgatives.—This may be ascribed to the volatility of the agent, which, diffusing itself through the colon, produced a general stimulation and contraction of the same, and being in the solid form, did not expend its volatile parts with so great a rapidity as to cause its premature expulsion, as occurs in the case of the fœtid enema. Hence in the numerous hysterical and dyspeptic affections, in which great annoyance is experienced from the pressure of an elastic tumor in the left hypochondrium, consisting of the arch of the colon distended by gas, beyond its capability of contraction, much benefit may be derived from the prolonged presence of the vapour of assafœtida, and from a perusal of the experiments related by Joerg† to have been performed with this substance, it appears the best adapted for producing an equal and universal contraction of the gut, the want of which is evidently a main cause of the distressing sensation just mentioned.

Suppositories consisting of one grain of elaterium in nine of soap, produced in two cases four dejections, in two caused one full dejection, and in one failed.

A suppository of two drops of croton oil added to soap, in a case of obstinate bowels, caused three full dejections, commencing in about ten minutes after its insertion. On a repetition, it caused the expulsion of one hard fæcal lump, and was followed by tenesmus.—In another case I formed a soap of croton oil and caustic potash, and having dissolved it in a large quantity of water, ordered it to be injected as an enema. Those observations, in connection with some made previously, tend to show that croton oil is not well suited for this purpose, and that if retained, it may cause serious irritation of the rectum.

*Dr. Osborne, in Dublin Journal.*

---

*Disease of the Posterior Columns of the spinal Cord, Sensation remaining unimpaired.*—Mr. Edward Stanley brought before the Royal Medical and Chirurgical Society of London, a well marked case of disease, strictly limited to the posterior columns of the spinal cord, yet producing phenomena at variance with the doctrine of the distinct influences of the anterior and posterior columns of the cord on the faculties of motion and sensation.

The disease, which was not the result of injury, commenced about three years before the patient's admission into St. Bartholomew's Hospital, with impaired motion of the lower extremities, at first slight, but progressively increasing, so that at the time of his admission he could only succeed by a great effort in raising his legs from the ground whilst sitting on a chair. Before death, the loss of motion became complete in each lower limb, throughout its whole extent. In no part, however, was there any defect of sensation, scratching, pricking, or pinching the skin, being attended by sensation as acute as in a sound limb.

On dissection, no signs of disease presented themselves excepting in the spinal chord. Here, contrary to the anticipations of many persons by whom this case was observed, no disease whatever was found in the anterior columns of the cord. An extensive change of structure and colour was, on the contrary, manifested in the posterior columns, from the pons to the lower end of the cord. "The value of this case, says the author, consists in the distinctness of its phenomena being acknowledged by many competent observers to have been such as they are recorded."

*Lond. Med. Gaz.* Feb. 7, 1840.

---

* Ganbii de Methodo concinnandi Formulus, Lug. Bat. 1767, p. 433.

† Joerg Materialen zu einer kunftigen Heilmittellehre, p. 366. This excellent work is wel named "Materials." I hope to make frequent use of the experimental portion as such, but while placing an implicit reliance on the facts, I shall have to offer different explanations of them from those given by the able author.

